# Hematological factors predicting mortality in patients with traumatic epidural or subdural hematoma undergoing emergency surgical evacuation: A retrospective cohort study

**DOI:** 10.1097/MD.0000000000022074

**Published:** 2020-09-11

**Authors:** Na Young Kim, Jaejoon Lim, Seunghoon Lee, Koeun Kim, Jung Hwa Hong, Duk-Hee Chun

**Affiliations:** a Department of Anesthesiology and Pain Medicine, Anesthesia and Pain Research Institute, Yonsei University College of Medicine, 50-1 Yonsei-ro, Seodaemun-gu, Seoul; b Department of Neurosurgery; c Department of Anesthesiology and Pain Medicine, CHA Bundang Medical Center, CHA University School of Medicine, 59 Yatap-ro, Bundang-gu, Seongnam; d Department of Policy Research Affairs National Health Insurance Service Ilsan Hospital, 100 Ilsan-ro, Ilsandong-gu, Goyang, Gyeonggi-do, Republic of Korea.

**Keywords:** epidural hematoma, emergency surgery, mortality, prognostic factor, subdural hematoma

## Abstract

Hematological abnormalities at admission are common after traumatic brain injuries and are associated with poor outcomes. The objective of this study was to identify the predictive factors of mortality among patients who underwent emergency surgery for the evacuation of epidural hematoma (EDH) or subdural hematoma (SDH).

This was a single-center retrospective cohort study of 200 patients who underwent emergency surgical evacuation of EDH or SDH between September 2010 and December 2018. Data on hematological parameters and clinical and intraoperative features were collected. The primary end-point was 1-year mortality after surgery. Univariate and multivariate analysis were performed, and the receiver operating characteristic (ROC) curves were assessed.

Of the 200 patients included in this study, 102 (51%) patients died within 1 year of emergency surgery. Lymphocyte count at admission, creatinine levels, activated partial thromboplastin time (aPTT), age, intraoperative epinephrine use, and Glasgow Coma Scale (GCS) score were significantly associated with mortality in the multivariate analysis. The areas under the ROC curve for the GCS score, aPTT, and lymphocyte counts were 0.677 (95% confidence interval [CI] 0.602–0.753), 0.644 (95% CI 0.567–0.721), and 0.576 (95% CI 0.496–0.656), respectively.

Patients with elevated lymphocyte counts on admission showed a higher rate of 1-year mortality following emergency craniectomy for EDH or SDH. In addition, prolonged aPTT and a lower GCS score were also related to poor survival.

## 1 Introduction

Mortality prediction on admission is valuable for effective perioperative management of patients with traumatic brain injury (TBI) undergoing surgery. Various factors at admission have been explored and identified as prognostic markers of TBI, such as Glasgow Coma Scale (GCS) score, absence of pupillary reactivity, international normalized ratio (INR), activated partial thromboplastin time (aPTT), platelet count, age, and neutrophil-to-lymphocyte ratio (NLR).^[1–3]^ However, previous studies on this topic included many different types of head injuries, and not all patients underwent surgery.

It is common for TBI patients to enter the operating room (OR) with only simple laboratory test results and without a thorough medical history evaluation or further advanced evaluation for emergency operation. It would be useful to find simple, quick, and accurate prognostic biomarkers that can be evaluated with hematological laboratory tests, which can predict the course of postoperative outcomes and guide treatment.

Posttraumatic neuroinflammation is a secondary injury after trauma that causes neurodegeneration and neurological impairment.^[4–8]^ The associations between poor prognoses in various types of cancer and inflammatory mediators have been well documented^[9–11]^; additionally, acute TBIs and inflammation are known to be associated.^[12–14]^ Recent studies have suggested that increased levels of inflammatory markers are the predictors of worse outcomes in acute neurosurgical emergencies, such as TBI, intracerebral hemorrhage (ICH), and subarachnoid hemorrhage (SAH).^[3,15,16]^ Additionally, INR, aPTT, and fibrinogen were found to be prognostic parameters in severe TBI.^[17]^ However, it is unclear which biomarkers are the best prognostic parameters for different severities and types of TBI.

This study aimed to evaluate traumatic epidural hematoma (EDH) or subdural hematoma (SDH) patients who presented to the OR for emergency evacuation of hematoma within 24 hours of injury to investigate hematological parameters on admission, including the coagulation profile and inflammatory biomarkers, to determine the prognostic factors associated with mortality 1 year after surgery.

## 2 Materials and methods

### 2.1 Patients

**Figure 1:**
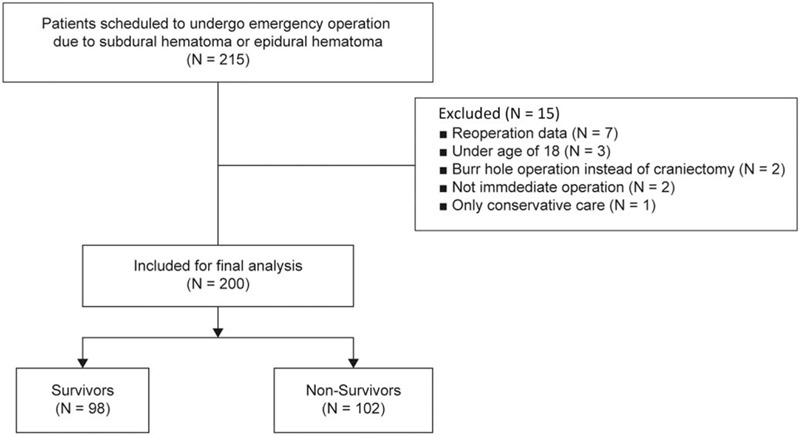
Flow diagram of patients.

This retrospective study included the electronic medical records of patients who underwent emergency evacuation for EDH or SDH at this institutional hospital. This study was approved by the Institutional Review Board and Hospital Research Ethics Committee (CHA Bundang Medical Center, Seongnam, Korea; IRB protocol No. 2019-02-037); the need for informed consent was waived due to the retrospective nature of this study. The data of 215 patients who underwent emergency surgery for EDH or SDH from September 2010 to December 2017 were assessed. Patients were included if they were admitted within 8 hours and underwent surgery within 24 hours of injury. Of the 215 patients identified initially, 15 were excluded for the following reasons: underwent reoperation (n = 7), under 18 years of age (n = 3), underwent burr hole surgery rather than craniectomy (n = 2), did not undergo surgery within 24 hours (n = 2), and only received conservative care (n = 1) (Fig. 1). Out of the remaining 200 patients, 165 patients with SDH and 35 patients with EDH were included in this study.

### 2.2 Data collection

All data from the electronic medical records were collected retrospectively. The following demographic and perioperative variables were evaluated: age, sex, height, weight, American Society of Anesthesiologists (ASA) physical status, GCS score, and the presence of underlying diseases such as hypertension, diabetes, cerebrovascular disease, and coronary and valvular heart disease. Also Charlson Comorbidity Index (CCI) score was calculated. Preoperative laboratory data included the white blood cell (WBC), platelet, neutrophil, and lymphocyte counts; hemoglobin, hematocrit, C-reactive protein, and creatinine (Cr) levels; red cell distribution width; mean platelet volume (MPV); aPTT; prothrombin time; and INR. Operative data included the operation and anesthesia times, intraoperative fluid inputs and outputs, transfusion amounts, and use of epinephrine. The preoperative NLR and platelet-to-lymphocyte ratio (PLR) were calculated. Finally, data on mortality 1 year following surgery were also collected.

### 2.3 Statistical analysis

Continuous variables were represented as means ± standard deviations (SDs) and were analyzed using the independent two sample t-test. Categorical variables were represented as the number (percentage) of patients and were analyzed using the chi-squared or Fisher exact tests.

For identifying the independent risk factors for 1-year mortality, univariate analysis was performed. The variables significantly associated with 1-year mortality (*P* value < .05) in the univariate analysis were then entered into the multivariate Cox regression analysis to identify the factors associated with 1-year mortality after adjustment for confounders. The results were represented as hazard ratios (HRs) and 95% confidence intervals (CIs). Receiver operating characteristic (ROC) curve analysis was also performed to evaluate the predictability of factors on admission. Results were represented as the area under the ROC curve (AUC) with 95% CIs. The Contal and O’Quigley method was used to predict the optimal cut-off values by maximizing the HRs. The Kaplan-Meier method was used to ascertain the survival curves 1 year after admission, and significance was determined using the log-rank test. SAS version 9.4 (SAS Institute INC., Cary, NC) was used for all statistical analyses. A *P* value < .05 was considered statistically significant.

## 3 Results

Table 1 presents the demographic and preoperative characteristics of the 200 patients who underwent emergency hematoma evacuation for EDH or SDH. Of the 200 patients, 102 (51%) died within 1 year of emergency surgery. Age, lymphocyte count, Cr level, aPTT, amount of intraoperatively administered packed red blood cells (pRBC), and number of patients receiving intraoperative epinephrine were significantly higher in the non-survival group than in the survival group. The mean lymphocyte count was 2.8 ± 2.0 in the survival group and 3.5 ± 2.4 in the non-survival group (*P* = .029). The mean Cr level was 0.9 ± 0.3 in the survival group and 1.3 ± 1.5 in the non-survival group (*P* = .014). In the survival and non-survival groups, the means of aPTT were 29.0 ± 4.6 and 33.0 ± 9.9, respectively (*P* < .001). Additionally, GCS scores and hematocrit levels were significantly lower in the non-survival group than in the survival group (*P* < .001 and *P* = .035, respectively). However, statistical differences were not found in the sizes of SDH or EDH hematoma (SDH [mm], 15.1 ± 8.7 vs 16.6 ± 8.8; EDH [mL], 83.7 ± 79.3 vs 107.3 ± 93.6), and other variables between the two groups.

**Table 1:**
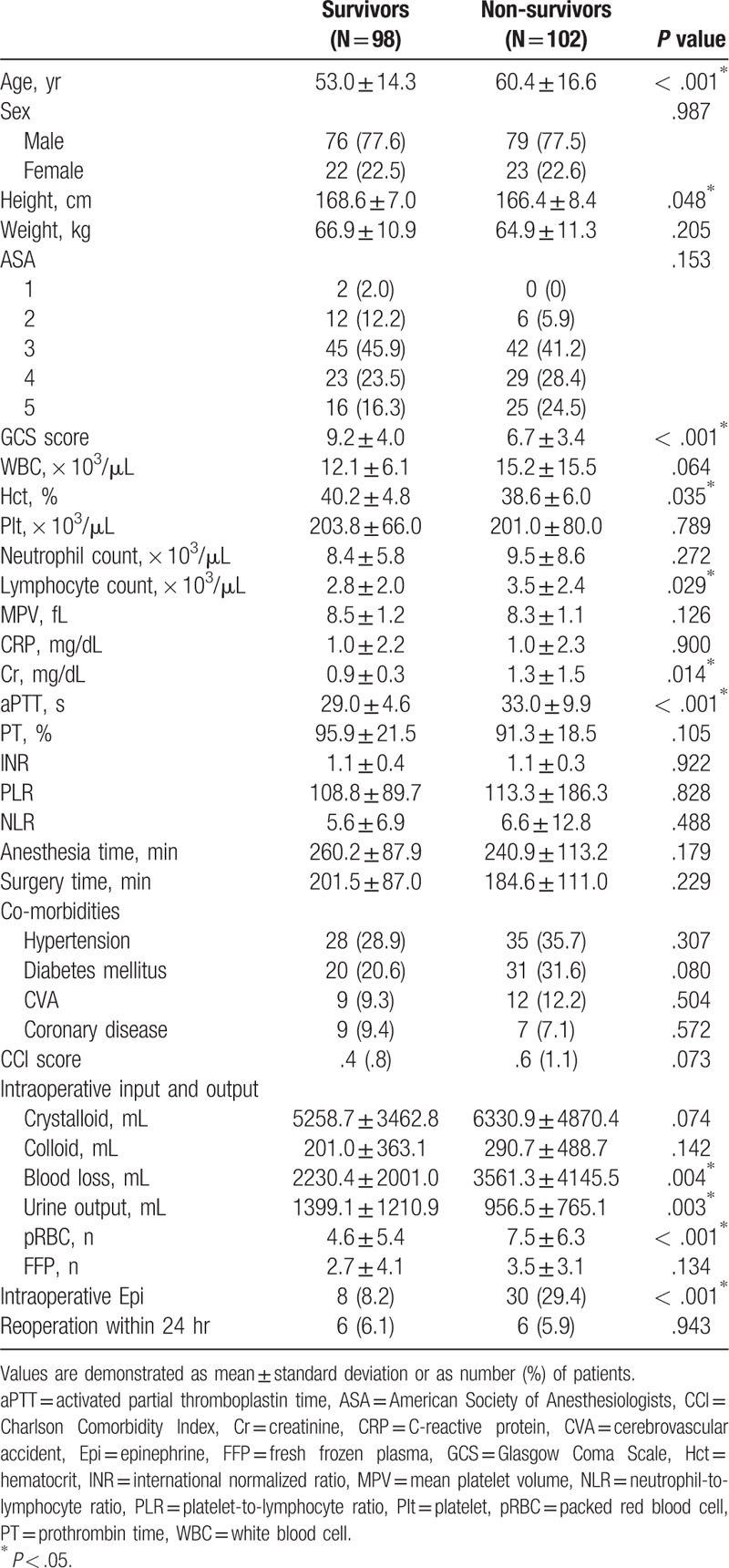
Demographic and preoperative characteristics.

The prognostic factors influencing 1-year mortality after emergency craniectomy are demonstrated in Table 2. On multivariate analysis, age (HR = 1.030, 95% CI = 1.015–1.046, *P* < .001), GCS score (HR = 0.884, 95% CI = 0.817–0.956, *P* = .002), Cr level (HR = 1.214, 95% CI = 1.039–1.418, *P* = .015), aPTT (HR = 1.045, 95% CI = 1.026–1.064, *P* < .001), the number of patients receiving intraoperative epinephrine (HR = 2.305, 95% CI = 1.396–3.806, *P* = .001), and lymphocyte count (HR = 1.085, 95% CI = 1.006–1.169, *P* = .034) were retained as significant predictors of 1-year mortality (Table 2).

**Table 2:**
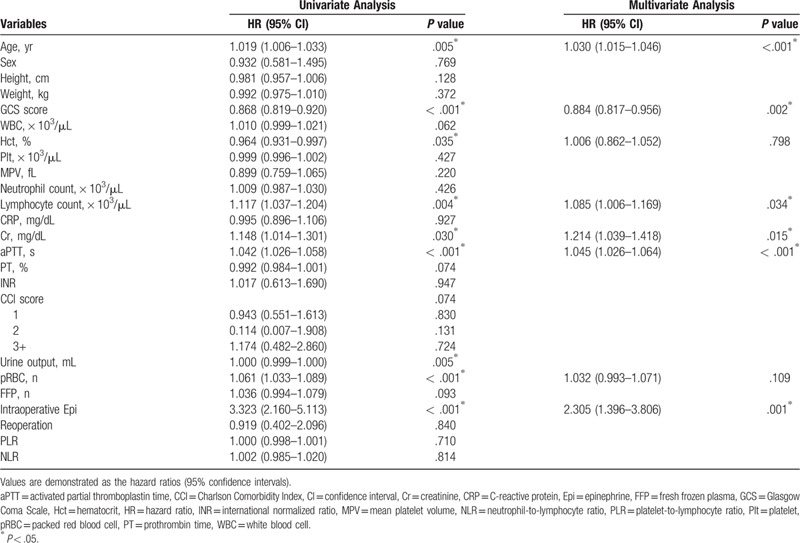
Univariate and multivariate analyses of prognostic factors for 1-year mortality after emergency craniectomy.

**Figure 2:**
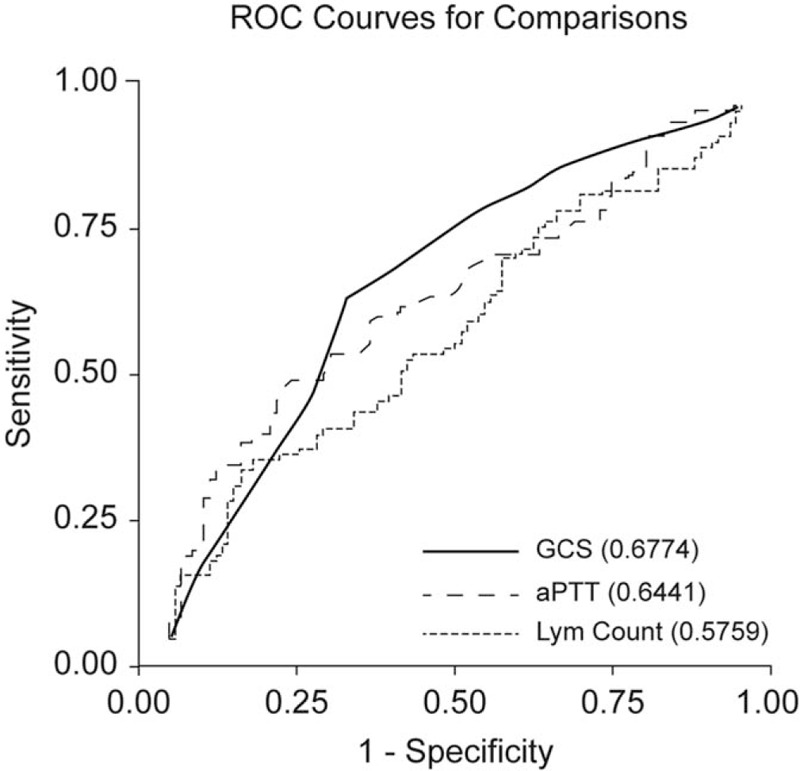
ROC curves of preoperative GCS scores, aPTT, and lymphocyte counts based on the multivariate logistic regression. ROC = receiver operating characteristics, GCS = Glasgow Coma Scale, aPTT = activated partial thromboplastin time.

Figure 2 demonstrates the ROC curves for GCS score, aPTT, and lymphocyte count based on multivariate logistic regression. The AUCs for GCS scores, aPTT, and lymphocyte counts were 0.677 (95% CI 0.602–0.753), 0.644 (95% CI 0.567–0.721), and 0.576 (95% CI 0.496–0.656), respectively.

**Figure 3:**
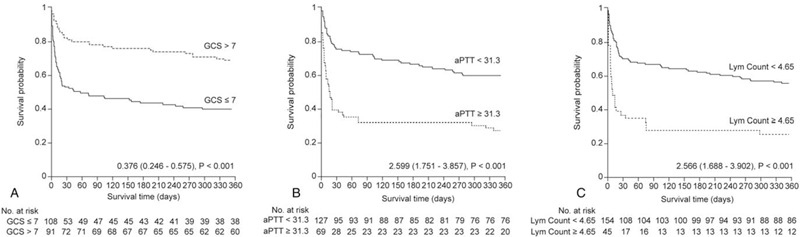
Kaplan-Meier curves for GCS scores, aPTT, and lymphocyte counts based on the optimal cut-off values. GCS = Glasgow Coma Scale, aPTT = activated partial thromboplastin time, Lym Count = lymphocyte count.

Figure 3 represents the Kaplan-Meier curves for GCS scores, aPTT, and lymphocyte counts according to cut-off values determined using the Contal and O’Quigley method, which is based on the log-rank test. We found that 1-year mortality after emergency craniectomy showed correlations with low GCS scores, elevated lymphocyte counts, and high aPTT.

## 4 Discussion

In this study, we investigated the prognostic value of hematological parameters on admission for predicting the 1-year mortality of patients who underwent emergency hematoma evacuation for EDH or SDH. We found that hematologic parameters, including aPTT, lymphocyte counts, and Cr levels, were higher among those who did not survive 1 year after surgery. Additionally, those who did not survive 1 year after surgery were older, had lower GCS scores on admission, and required intraoperative epinephrine use more frequently.

Inflammation is an important injury mechanism that affects neurodegeneration and induces neurological impairments, leading to poor outcomes in TBI patients.^[18]^ Mechanical injury and brain tissue damage activate the peripheral immune system, and secondary brain injuries are caused by acute inflammatory responses in patients with TBI and ICH.^[19,20]^ Such excessive immune responses are responsible for increased severity of brain tissue injuries and poor outcomes.^[19,20]^ This suggests that the peripheral blood immune responses can be used as prognostic factors for acute brain injuries. NLR, PLR, and MPV are known to be associated with inflammation,^[9,11,21]^ and NLR has been investigated as a prognostic factor for TBI and other neurosurgical emergencies.^[3,16,22]^ Therefore, we investigated the association between hematologic parameters, including inflammatory indices, and mortality among EDH or SDH patients undergoing emergency surgery. NLR, PLR, and MPV values at admission were calculated and compared; however, there was no difference between survivors and non-survivors. Additionally, the NLR did not increase considerably and was lower than that reported in other studies that found a correlation between increased NLR at admission and unfavorable outcomes.^[3,23]^ Prognostic factors and their values may vary depending on the type of injury since TBI involves a diverse group of brain injuries that differ in cause, severity, and pathogenesis. For example, NLR at admission was not associated with 3-month mortality in patients with acute ICH, even though it was associated with more serious stroke.^[24]^ Furthermore, the NLR in ICH patients with an unfavorable outcome^[16,22]^ was much lower than that reported in TBI studies.^[3,23]^ NLR was not found to have predictive capacity for mortality in this study presumably because we only included EDH or SDH patients.

Changes in lymphocyte counts during the course of ICH have been reported. Admission lymphocytopenia^[25,26]^ as well as the development of lymphocytopenia during treatment were related to an unfavorable outcome.^[25]^ Lymphocyte dynamics during the course of TBI have been studied previously. In a pilot study, Petrone et al^[27]^ investigated the lymphocyte and neutrophil dynamics following TBI based on severity and different time points after injury. They found that severe TBI patients had higher lymphocyte and lower neutrophil percentages than mild TBI patients from 0 to 6 hours and up to 24 hours post-TBI. This relationship reversed 48 hours after TBI. In our study, non-survivors had higher lymphocyte counts on admission than survivors. Additionally, non-survivors had lower GCS scores. These high lymphocyte counts and low GCS scores indicate that trauma was more severe in non-survivors. After surgery, patients with poor outcomes might develop lymphocytopenia due to the course of the disease itself. Nevertheless, elevated admission lymphocyte counts (≥ 4.65 10^3^/μL [Fig. 3]) are useful for the prognosis of mortality because they can differentiate patients according to the severity of trauma and outcomes upon admission.

Factors such as age, GCS scores, and aPTT were also found to have prognostic value in patients with EDH or SDH. These results were consistent with those from other studies.^[2,28]^ The GCS score is one of the most commonly used predictors of outcomes^[29]^; additionally, increasing age is known to be associated with higher mortality after TBI.^[30,31]^ Coagulopathy is common in patients with severe TBI, and the incidence of coagulopathy increases with TBI severity.^[32,33]^ Studies have found that abnormal values for coagulation parameters such as INR, aPTT, and fibrinogen were associated with unfavorable outcomes after TBI.^[17,28]^ Mortality prediction on admission is important for effective intra- and post-operative patient management. Data on the significant prognostic factors identified in this study can be readily obtained upon admission to stratify patients according to risk profile and to assist in patient management.

This study had certain limitations that should be acknowledged. This was a single-center, retrospective study that derived data solely from electronic medical records; thus, it is susceptible to bias and other confounding factors. In addition, the follow-up period after surgery was very short. However, this study is valuable in that it focuses on admission laboratory data for predicting the prognosis of traumatic EDH or SDH patients, and this could assist in making clinical decisions during the perioperative period.

In conclusion, patients with elevated lymphocyte counts on admission showed a higher rate of 1-year mortality following emergency craniectomy for EDH or SDH. Additionally, prolonged aPTT and low GCS scores were also related to poor survival. These results may be useful for predicting the prognosis and for determining the direction of perioperative management in these patients.

## Author contributions

**Conceptualization:** Jaejoon Lim, Duk-Hee Chun.

**Data curation:** Na Young Kim, Seunghoon Lee.

**Formal analysis:** Jung Hwa Hong.

**Investigation:** Na Young Kim, Jaejoon Lim, Seunghoon Lee, Koeun Kim, Duk-Hee Chun.

**Methodology:** Na Young Kim, Koeun Kim.

**Project administration:** Duk-Hee Chun.

**Supervision:** Na Young Kim.

**Validation:** Jaejoon Lim, Duk-Hee Chun.

**Writing – original draft:** Na Young Kim, Duk-Hee Chun.

**Writing – review & editing:** Duk-Hee Chun.
